# New semi‐dwarfing alleles with increased coleoptile length by gene editing of *gibberellin 3‐oxidase 1* using CRISPR‐Cas9 in barley (*Hordeum vulgare* L.)

**DOI:** 10.1111/pbi.13998

**Published:** 2023-02-08

**Authors:** Jingye Cheng, Camilla Hill, Yong Han, Tianhua He, Xingguo Ye, Sergey Shabala, Ganggang Guo, Meixue Zhou, Ke Wang, Chengdao Li

**Affiliations:** ^1^ Tasmanian Institute of Agriculture University of Tasmania Hobart TAS Australia; ^2^ Western Crop Genetics Alliance, Food Futures Institute, College of Science, Health, Engineering and Education Murdoch University Murdoch WA Australia; ^3^ Agriculture and Food, Department of Primary Industries and Regional Development South Perth WA Australia; ^4^ Institute of Crop Science Chinese Academy of Agricultural Sciences Beijing China; ^5^ School of Biological Science University of Western Australia Perth WA Australia

**Keywords:** semi‐dwarfing gene, plant height, coleoptile, flowering transition, CRISPR/Cas9, gibberellin

## Abstract

The green revolution was based on genetic modification of the gibberellin (GA) hormone system with “dwarfing” gene mutations that reduces GA signals, conferring shorter stature, thus enabling plant adaptation to modern farming conditions. Strong GA‐related mutants with shorter stature often have reduced coleoptile length, discounting yield gain due to their unsatisfactory seedling emergence under drought conditions. Here we present *gibberellin* (*GA*) *3‐oxidase1* (*GA3ox1*) as an alternative semi‐dwarfing gene in barley that combines an optimal reduction in plant height without restricting coleoptile and seedling growth. Using large‐scale field trials with an extensive collection of barley accessions, we showed that a natural *GA3ox1* haplotype moderately reduced plant height by 5–10 cm. We used CRISPR/Cas9 technology, generated several novel *GA3ox1* mutants and validated the function of *GA3ox1*. We showed that altered *GA3ox1* activities changed the level of active GA isoforms and consequently increased coleoptile length by an average of 8.2 mm, which could provide essential adaptation to maintain yield under climate change. We revealed that CRISPR/Cas9‐induced *GA3ox1* mutations increased seed dormancy to an ideal level that could benefit the malting industry. We conclude that selecting *HvGA3ox1* alleles offers a new opportunity for developing barley varieties with optimal stature, longer coleoptile and additional agronomic traits.

## Introduction

As a key part of the modern agricultural revolution, the modification of gibberellin (GA) genes led to the development of semi‐dwarf varieties with reduced plant height and enhanced yield (Eshed and Lippman, 2019). The most widely used semi‐dwarfing genes in barley, rice and wheat reduce plant height through reduced sensitivity to the growth hormone gibberellic acid (GA) (Hedden, 2003; Peng *et al*., 1999). Shorter stems reduce competition for resources and can increase assimilate partitioning to the developing grains – improving harvest index, floret fertility and yield while reducing the risk of lodging (Pearce, 2021).

Several studies have also reported adverse effects on the agronomic performance of GA‐related semi‐dwarfing genes (Chen *et al*., 2019). For example, some semi‐dwarfing genes were found to overly reduce plant height, making mechanical harvest difficult (Carter *et al*., 2019) and less economically profitable in areas where straw is an essential commodity as animal feed (Annicchiarico and Pecetti, 2003). Other studies reported reductions in grain weight, yield and biomass, particularly in droughted growing environments (e.g. Jatayev *et al*., 2020). In warm and dry environments, commonly found in Australia's cropping regions and Mediterranean climates, cereal seeds are sown deeply due to limited soil moisture (Zhao *et al*., 2022). Semi‐dwarfing genes have been reported to decrease coleoptile length, leading to poor emergence under deep sowing (Rebetzke *et al*., 2013; Trethowan *et al*., 2001). GA also regulates flowering in cereal crops (King and Evans, 2003). Reduced GA signals delay flowering (Boden *et al*., 2014), which can negatively impact yield (He *et al*., 2021). As climate change affects the intensity and frequency of precipitation, there is an urgent need for plant breeders to select short‐statured barley cultivars with enhanced early emergence characteristics. Currently, little information is available on alternative semi‐dwarfing genes and alleles in barley that, in combination, provide an optimal reduction in plant height without restricting coleoptile and seedling growth and flowering transition.

There are four major semi‐dwarfing genes used in barley breeding programmes. S*hort culm 1* (*hcm1*), located on chromosome 2HL, is present in USA's six‐rowed barley and leads to a height reduction of 10 cm (Franckowiak, 2000; Yu *et al*., 2010). *Semi‐brachytic 1* (*uzu*) is used in East Asian barley breeding programmes, is located on chromosome 3HL, and is characterized by a missense mutation caused by a single nucleotide substitution in the brassinolide‐responsive gene *HvBRI1* (Chono *et al*., 2003; Jing, 2000). *Breviaristatum‐e* (*ari‐e*) is located on chromosome 5HL and is the most widely used semi‐dwarfing gene in spring European cultivars (Ellis *et al*., 2002). The *semi‐dwarf 1* (*sdw1*) locus is the most commonly used semi‐dwarfing gene in Asia, North and South America, Europe and Australia. *sdw1* is an ortholog of the rice *sd1* gene (Jia *et al*., 2009). In barley, *sdw1* corresponds to the gene *HvGA20ox2* encoding the gibberellin 20‐oxidase (Xu *et al*., 2017). Barley plants with the homozygous *sdw1* gene are 20 to 30 cm *shorter than wild types* (Bélanger *et al*., 2018; Jia *et al*., 2009; Xu *et al*., 2017). The allele *sdw1.d* contains a 7 bp deletion in exon1 of *HvGA20ox2* and confers a shorter and stronger stalk by reducing biosynthesis, but also delaying flowering, reducing seedling establishment and vigour due to shorter coleoptiles (Paynter and Clarke, 2010; Teplyakova *et al*., 2017).

In addition to GA20ox, gibberellin (GA)‐3 oxidase (GA3ox) is an important 2‐oxoglutarate‐dependent dioxygenase for catalysing GA biosynthesis (Hedden and Thomas, 2012; Mitchum *et al*., 2006; Sun and Gubler, 2004). Previous research identified significant genetic variants at *HvGA3‐oxidase1* (HORVU.MOREX.r3.2HG0208900.1) are associated with flowering transition, plant height and grain yield in barley (He *et al*., 2019; Hill *et al*., 2019a,b).

Gene‐editing techniques can introduce precise and predictable gene modifications into crop plants to obtain desired traits (Gao, 2021). Reports on the successful deployment of CRISPR/Cas9 technology in the barley genome are emerging. For example, CRISPR/Cas9 technology targeted *HvPM19*, a gene encoding a plasma membrane protein that regulates grain dormancy, and *Protein Targeting to Starch 1* (*ptst1*) involved in starch accumulation that regulates germination (Lawrenson *et al*., 2015; Zhong *et al*., 2019), and successfully created mutants with improved grain quality and nutrition. Allelic mutants of *starch synthase IIa* (*SSIIa*) created by CRISPR/Cas9 showed increased amylose and resistance starch (RS) contents and improved resistance to starch digestion (Yang *et al*., 2022). Gene‐editing tools such as CRISPR/Cas9 can enhance allelic diversity by directly engineering genetic variation in target genes (Zhang *et al*., 2020), broadening genetic diversity to fine‐tune traits such as plant height and flowering transition.

In this study, we precisely edited *HvGA3ox1* in the Western Australian two‐rowed spring malting barley cultivar Vlamingh. We created novel semi‐dwarfing genes that confer optimized plant height and coleoptile length without adversely affecting other important agronomic traits. Genetic variants within *HvGA3ox1* were characterized to define and identify functional haplotypes associated with flowering time, plant height and grain yield components. This study provides opportunities for plant breeders to select short‐statured barley with improved seedling emergence characteristics and agronomic performance. Such barley can offer significant advantages in Mediterranean growing environments where deep sowing into stored soil moisture is required.

## Results

### Genetic and haplotype variation of *HvGA3ox1*

*HvGA3ox1* is a 2.938 kb gene in length located on chromosome 2H and is annotated as encoding gibberellin 3 beta‐hydroxylase, a key enzyme in the gibberellin synthesis pathway.

**Figure 1:**
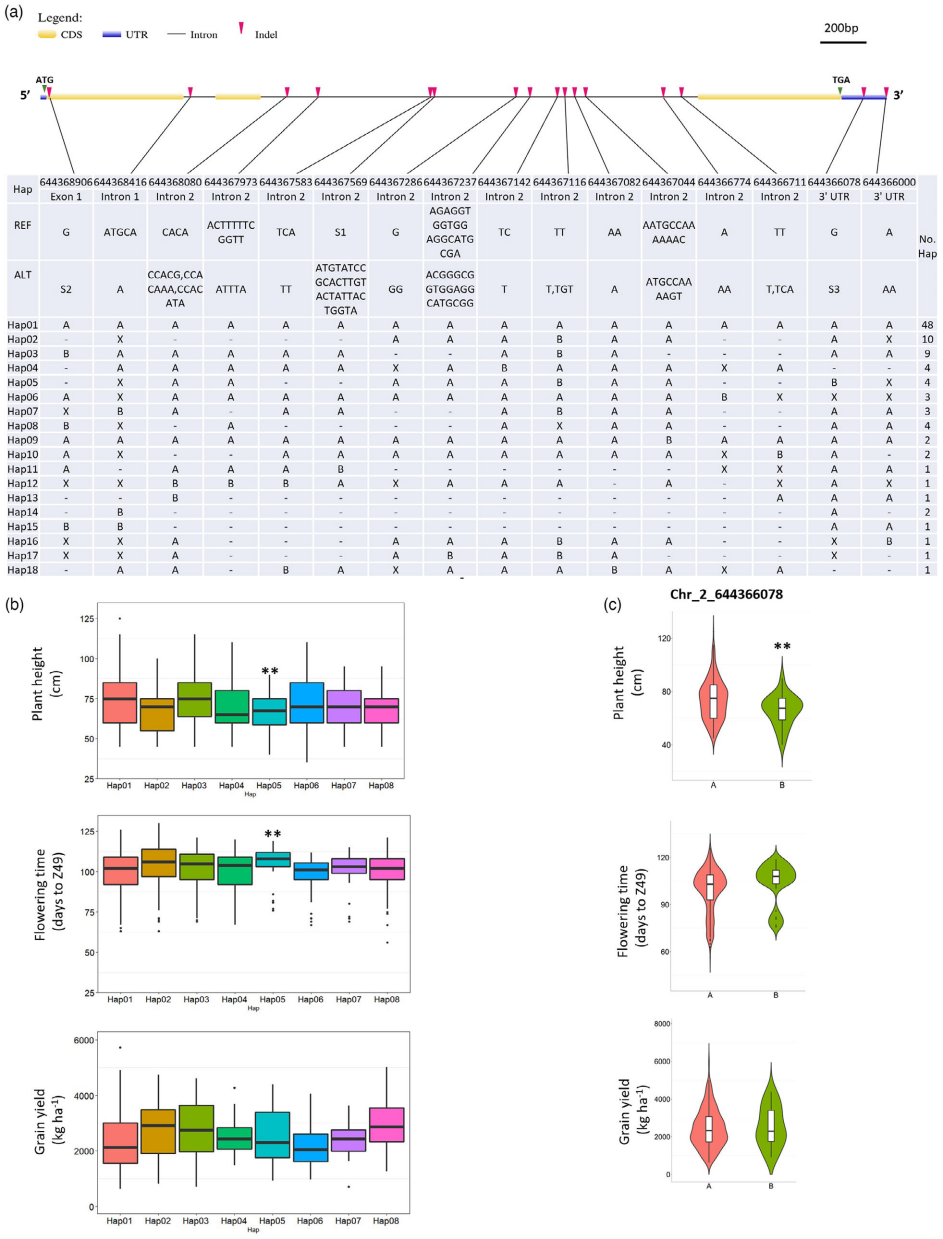
Haplotypes based on insertion–deletion polymorphisms in *HvGA3ox1* across the 632 barley accessions and the distribution of 8 major haplotypes as well as functional polymorphism in days to Z49, plant height (PH) and grain yield (GY). (a) The type of polymorphism, the position of polymorphic nucleotides and the reference/alternative alleles are present under each InDel. “A” indicates reference allele; “B” indicates alternative allele; “X” indicates heterozygous allele; “_” indicates missing data; “UTR” indicates the untranslated region. “CDS” indicates coding sequence. “ATG” and “TGA” indicate the start and stop codons; “No. Hap” indicates the number of each haplotype. Sequences of “S1”, “S2” and “S3” were shown in Table S1. (b) The effect of each haplotype on flowering transition (days to Z49), plant height (PH), and grain yield (GY). “Hap” indicates haplotype; Hap05 had significantly lower plant height and longer days to Z49, compared to the reference Hap01. (c) PH, days to Z49 and GY variation between different genotypes for Chr_2_644366078, showing B group had significantly lower PH than A group; A = G; B = GCCTGCTACCGCTTGTCGTG, GCCTGCTAACGCTTGTCGTG, GCTTGCTACCGCTTGTCGTG. *Note*: **P value <0.01, as determined by Student's *t*‐test. “PH” indicates plant height; “GY” indicates grain yield.

Two GA3ox‐related genes, *HvGA3ox1* and *HvGA3ox2*, were previously described in barley (Spielmeyer *et al*., 2004). *HvGA3ox1* is closely related to TraesCS2B03G1429000 in wheat (Figure S1). The duplication of *GA3ox1* from *GA3ox2* occurred in the common ancestor of Triticeae species (Figure S1). From the analysis of *HvGA3ox1* gene sequence in a panel of 632 barley accessions (Hill *et al*., 2021), we identified 52 genetic variants including 36 single nucleotide polymorphisms (SNPs) and 16 insertion/deletion polymorphisms (InDels). Among these, three SNPs caused amino acid change, leading to missense variants (Table S1). Sixteen InDels grouped *HvGA3ox1* into eight haplotypes (with haplotype frequency >2%) (Figure 1a). We evaluated the haplotype significance and effects based on three agronomic traits: flowering time (days to Zadoks development stage 49, Z49), plant height (PH) and grain yield (GY). One functional haplotype (Hap05) was significantly associated with the delayed flowering time and shorter plant stature compared to the reference haplotype 01 (Hap01) (Figure 1b). Hap05 was distinguished from other haplotypes by containing an insertion polymorphism at Chr_2_644366078. This 19 bp insertion is closely linked to an SNP (Chr_2_644367121) that was identified as significantly associated with the flowering time (Hill *et al*., 2019a) (Table S2).

### Generation of barley *ga3ox1* mutants by CRISPR/Cas9‐mediated gene editing

**Figure 2:**
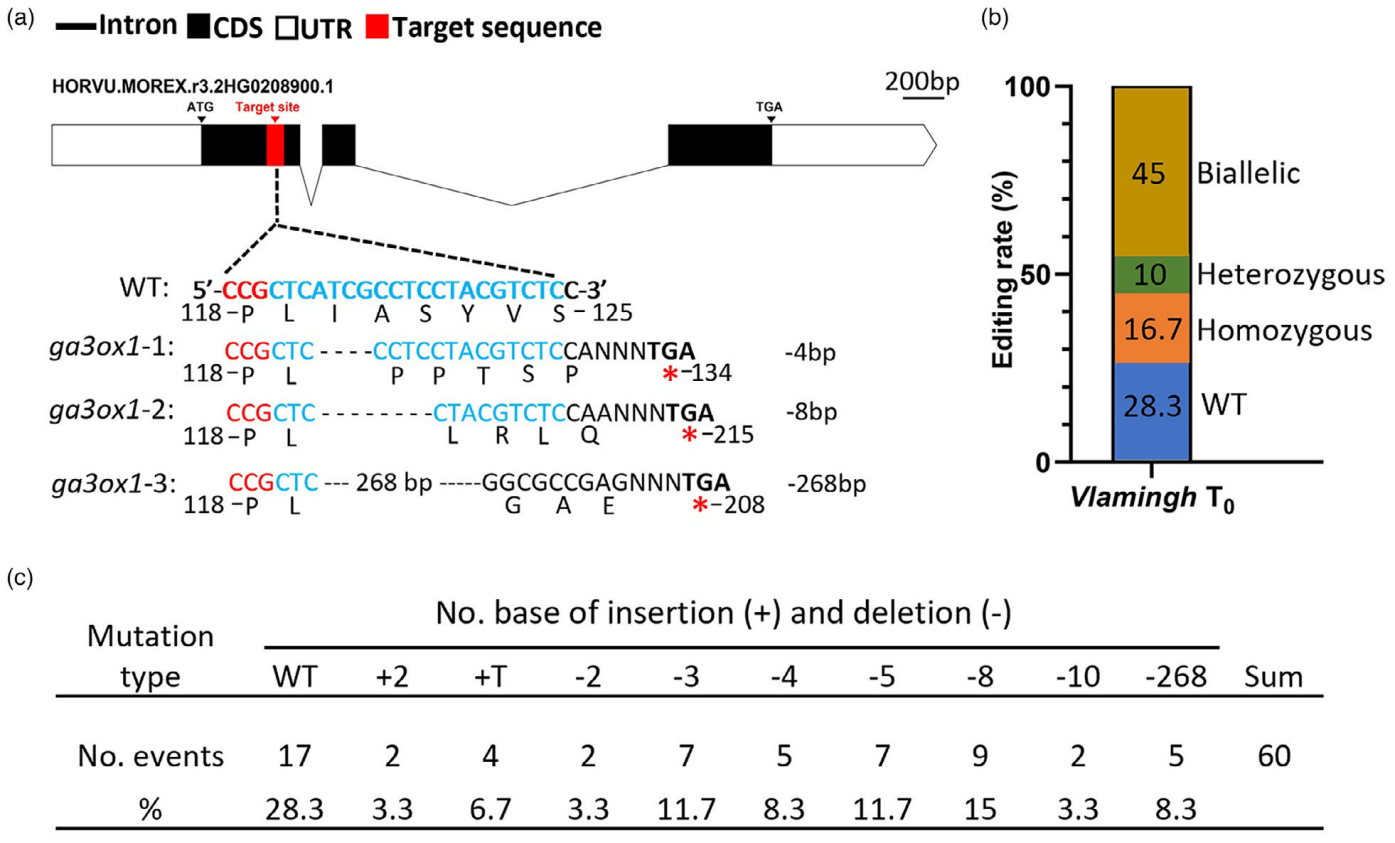
Gene structure of *HvGA3ox1* and the editing rate of the donor variety Vlamingh and all mutation types in *HvGA3ox1 T*_0_ edited plants generated by CRISPR/Cas9 technology. (a) Schematic diagram of gene structure and single guide RNA target site in *HvGA3ox1* (HORVU.MOREX.r3.2HG0208900) as well as the changes in the DNA sequence and amino acid sequence of the targeted region in *ga3ox1‐1*, *ga3ox1‐2* and *ga3ox1‐3*. The target sequence is shown in blue with the protospacer adjacent motif highlighted in red. ATG and TGA are the start and stop codons, respectively. (b) Rate for different genotypes including homozygous, heterozygous, biallelic mutations, and WT by editing the donor variety Vlamingh using CRISPR/Cas9. (c) All CRISPR/Cas9‐mediated knockout mutations in *HvGA3ox1 T*_0_ edited plants. *Note*: “WT” indicates wild type.

We used CRISPR/Cas9‐mediated gene editing to determine the effect of *HvGA3ox1* on plant height and other related agronomic traits. *HvGA3ox1* consists of three exons and two introns and encodes a 378 amino acid protein (Figure 2a). We have designed one unique single guide RNA (sgRNA) in the exon1 of *HvGA3ox1*, on the antisense strand with a GC content of 65.2% (Figure 2a). A total of 60 *T*_0_ plants from the Western Australian malting barley cultivar “Vlamingh” were successfully regenerated by *Agrobacterium*‐mediated transformation methods (Zang *et al*., 2022). Using polymerase chain reaction (PCR) with *HvGA3ox1*‐specific primers and subsequent Sanger sequencing, 17 wild‐type (28.3%), six heterozygous (10%), 27 biallelic (45%, two DNA strands have different mutations) and ten homozygous (16.7%) mutations were detected (Figure 2b). We identified nine mutation types in the 60 *T*_0_ events, which varied from 1 bp to 268 bp insertion or deletion (Figure 2c). The majority of the mutations were 3–8 bp deletions (46%).

Three *T*_3_ homozygous mutant transgenic lines originated from *HvGA3ox1*‐knockout plants, including *ga3ox1‐1*, *ga3ox1‐2* and *ga3ox1‐3* were selected for further analysis. *ga3ox1‐1*, *ga3ox1‐2* and *ga3ox1‐3* contained a 4 bp, 8 bp and 268 bp deletion, respectively. The deletions caused a frameshift and introduced early stop codons, leading to the production of truncated proteins (Figure 2a). For example, the 4, 8 and 268 bp deletions in *ga3ox1‐1* resulted in frameshift mutations introducing premature stop codons and initiating termination at 134th, 215th and 208th amino acid, respectively.

### Characterization of *ga3ox1* mutants shows differences in flowering time and plant height compared to the transgenic null line

**Figure 3:**
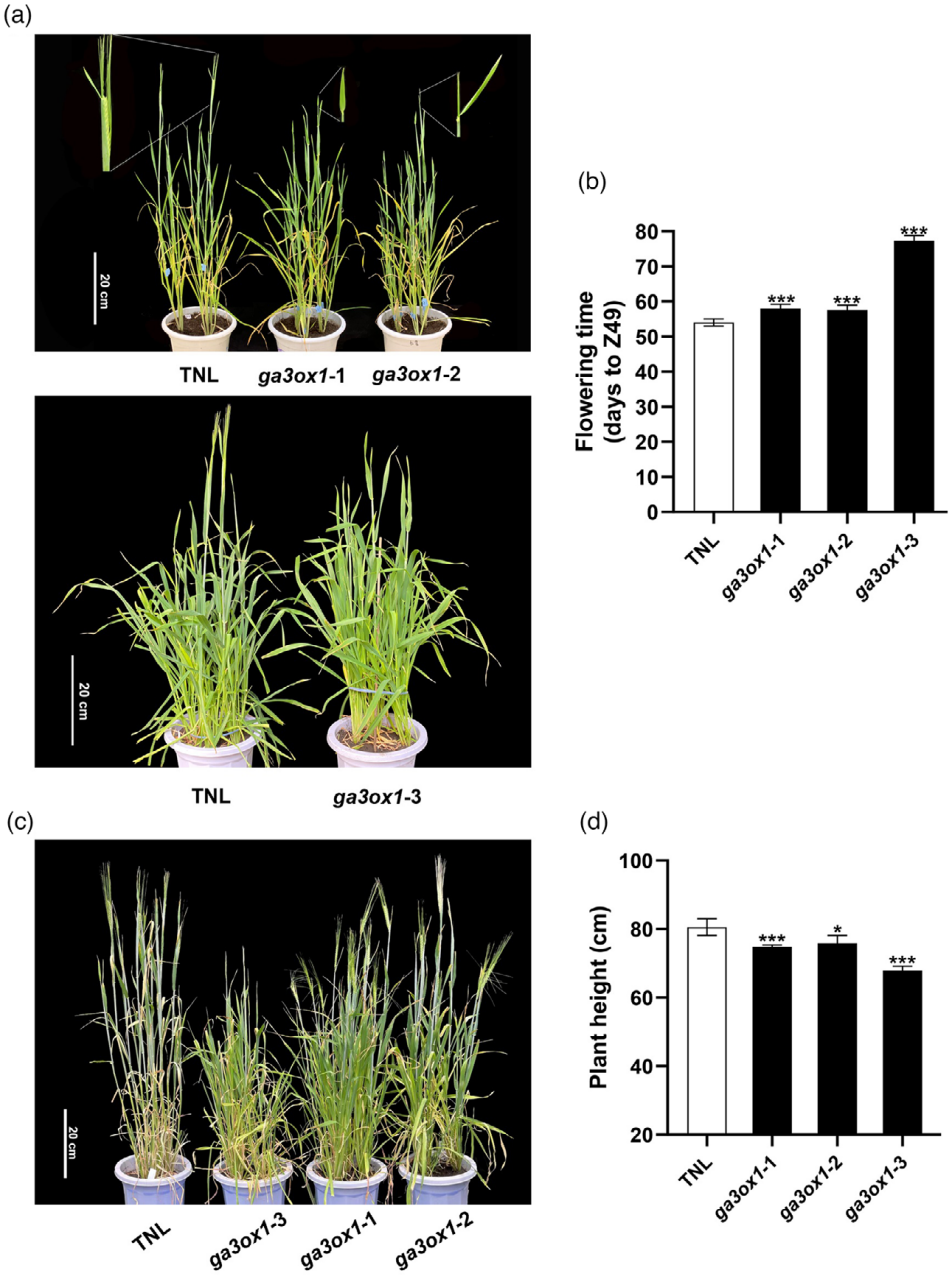
Flowering time and plant height of barley *ga3ox1* mutants. (a) Morphology of the *ga3ox1* mutants and transgenic null line (TNL) on days to Z49. (b) Comparison of days to Z49 between *ga3ox1* mutants and TNL. (c) Morphology of *ga3ox1* mutants and TNL on plant height (PH). (d) Comparison of PH between *ga3ox1* mutants and TNL. *Note*: Error bars indicate standard deviation among three biological replicates. *P < 0.05, **P < 0.01, ***P < 0.001, ns, not significant, as determined by Student's *t*‐test.

**Figure 4:**
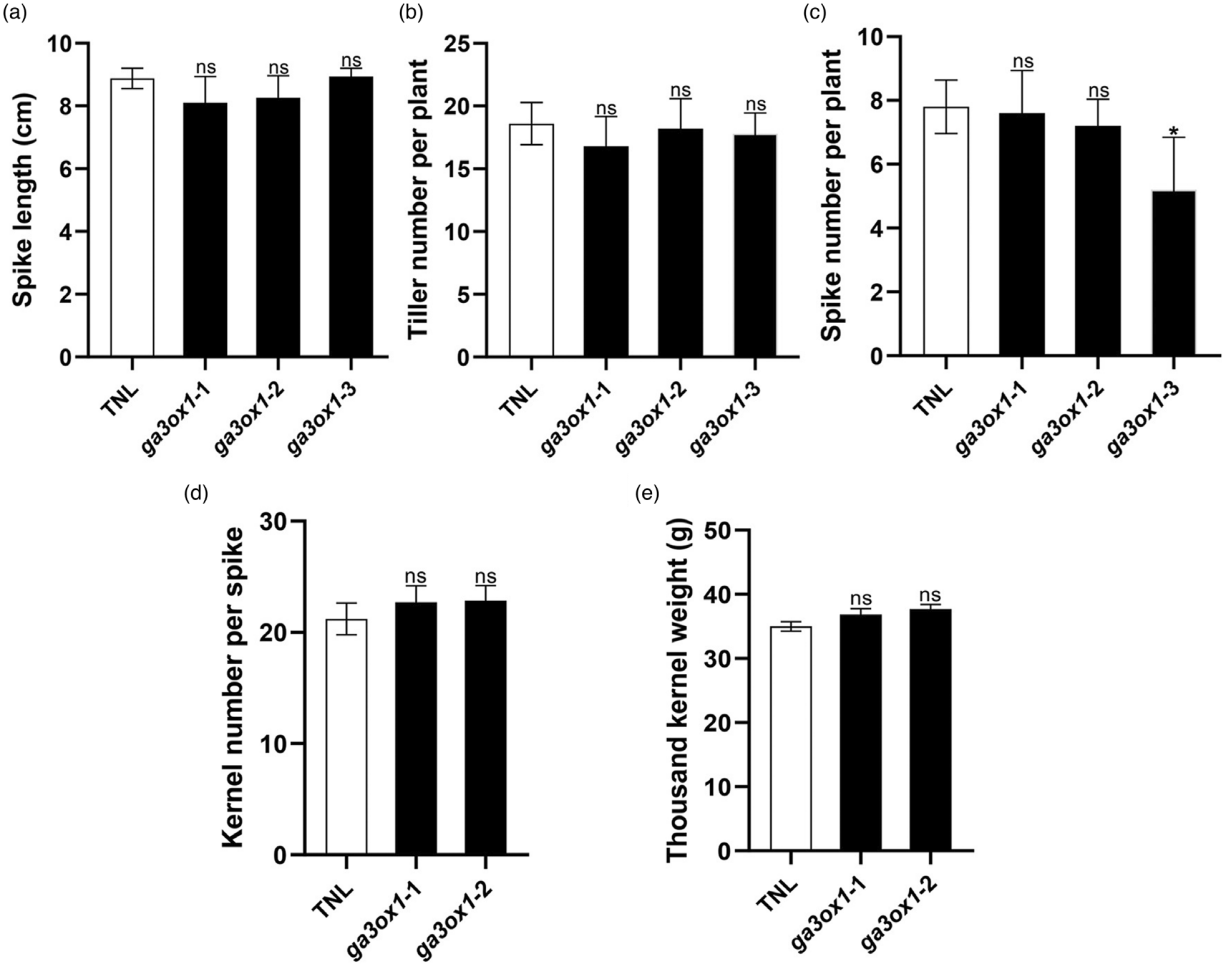
Impact of the disruption to *HvGA3ox1* to grain yield and associated traits in barley *ga3ox1* mutants. (a) Spike length (cm), (b) Tiller number per plant, (c) Spike number per plant, (d) Kernel number per spike and (e) Thousand kernel weight (g) of the *ga3ox1* mutants and TNL. *Note*: Error bars indicate SD among three biological replicates. *P < 0.05, **P < 0.01, ***P < 0.001, ns, not significant, as determined by Student's *t*‐test. “GY” indicates grain yield; “TNL” indicates transgenic null line.

Five replicates from the three selected mutant lines were used to evaluate the different agronomically relevant traits, including flowering time, plant height (PH), spike length, spike number per plant, kernel number per spike, tiller number and 1000 kernel weight (TKW). The three T3 homozygous mutant lines exhibited distinct phenotypes when compared to the transgenic null line (TNL). Mutant lines *ga3ox1‐1* and *ga3ox1‐2* exhibited delayed flowering time by 4 days, while the *ga3ox1‐3* mutant delayed flowering time by 19 days (Figure 3a,b). PH was measured at mature plants. Compared to TNL, *ga3ox1‐1* and *ga3ox1‐2* mutants had reduced PH with an average reduction of 5.3 cm. The *ga3ox1‐3* mutation reduced 12.7 cm in PH (Figure 3c,d). However, no significant differences between TNL and *ga3ox1* mutants were detected for spike length, tiller number per plant, spike number per plant, kernel number per spike and TKW, except for the *ga3ox1‐3* mutant which had significantly lower spike numbers per plant (Figure 4).

### Barley *ga3ox1* mutants have improved seed dormancy and longer coleoptiles

**Figure 5:**
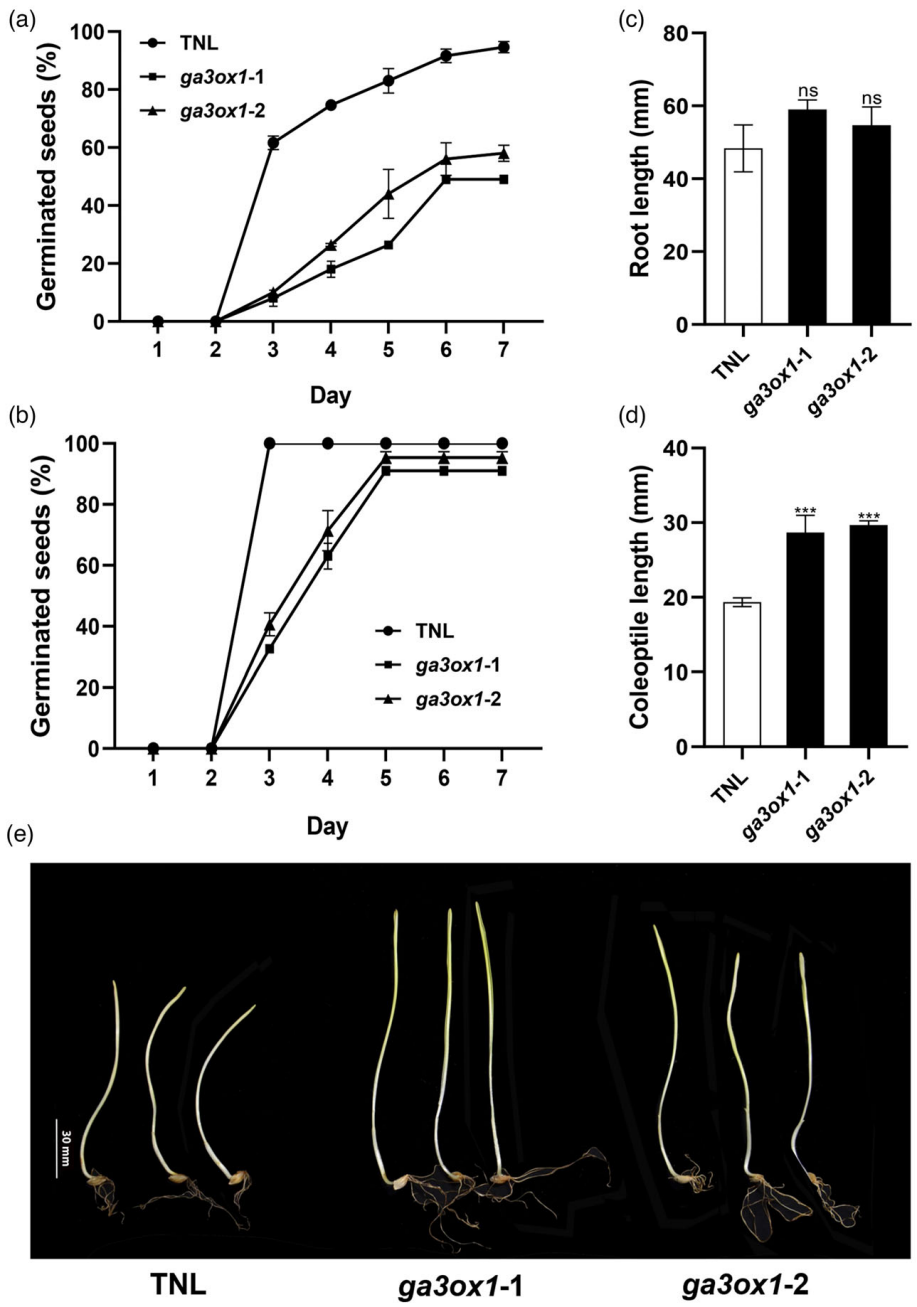
Comparison of germinated rate, root length and coleoptile length between *ga3ox1* mutants and TNL. The germination rate (%) of the 50 seeds that were (a) 10 days after harvest and (b) 2 months after harvest were compared between *ga3ox1* mutants and transgenic null line (TNL). Seven days after germination, (c) Root length (cm) and (d) coleoptile length (cm) compared between *ga3ox1* mutants and TNL. (e) Morphology of the *ga3ox1* mutants and TNL on coleoptile length. *Note*: Error bars indicate standard deviation among three biological replicates. ***P < 0.001, ns, not significant, as determined by Student's *t*‐test. “TNL” indicates transgenic null line.

We tested the seed germination of two *ga3ox1* mutants. The germination rate of *ga3ox1‐1* and *ga3ox1‐2* mutant seeds was delayed by 4 days to reach 60% germination rate compared with that of the TNL seeds 10 days after harvest (Figure 5a). After 2 months, the dormancy in barley seeds was significantly decreased. The germination rate of *ga3ox1* mutants was delayed by only 1 day to reach 60%, and by 3 days to reach 90% of germination rate, compared with that of the TNL (Figure 5b). Despite the delay, *ga3ox1* mutants reached a similar level of germination rate compared with the TNL. CRISPR/Cas9‐mediated knockout *HvGA3ox1* increased the seed dormancy period but maintained the intrinsic germinative vigour. We examined the effect of *ga3ox1* mutations on root length and coleoptile length. Root length and coleoptile length of seedlings of *ga3ox1* mutants and TNL barley were measured 7 days after germination. The two *ga3ox1* mutants, *ga3ox1‐1* and *ga3ox1‐2*, had no detectable effects on root length (Figure 5c). However, both mutations increased coleoptile length by an average of 8.2 mm compared with TNL (Figure 5d).

### Reduced contents of bioactive GA isoforms in the developing grains of *ga3ox1* mutants

**Figure 6:**
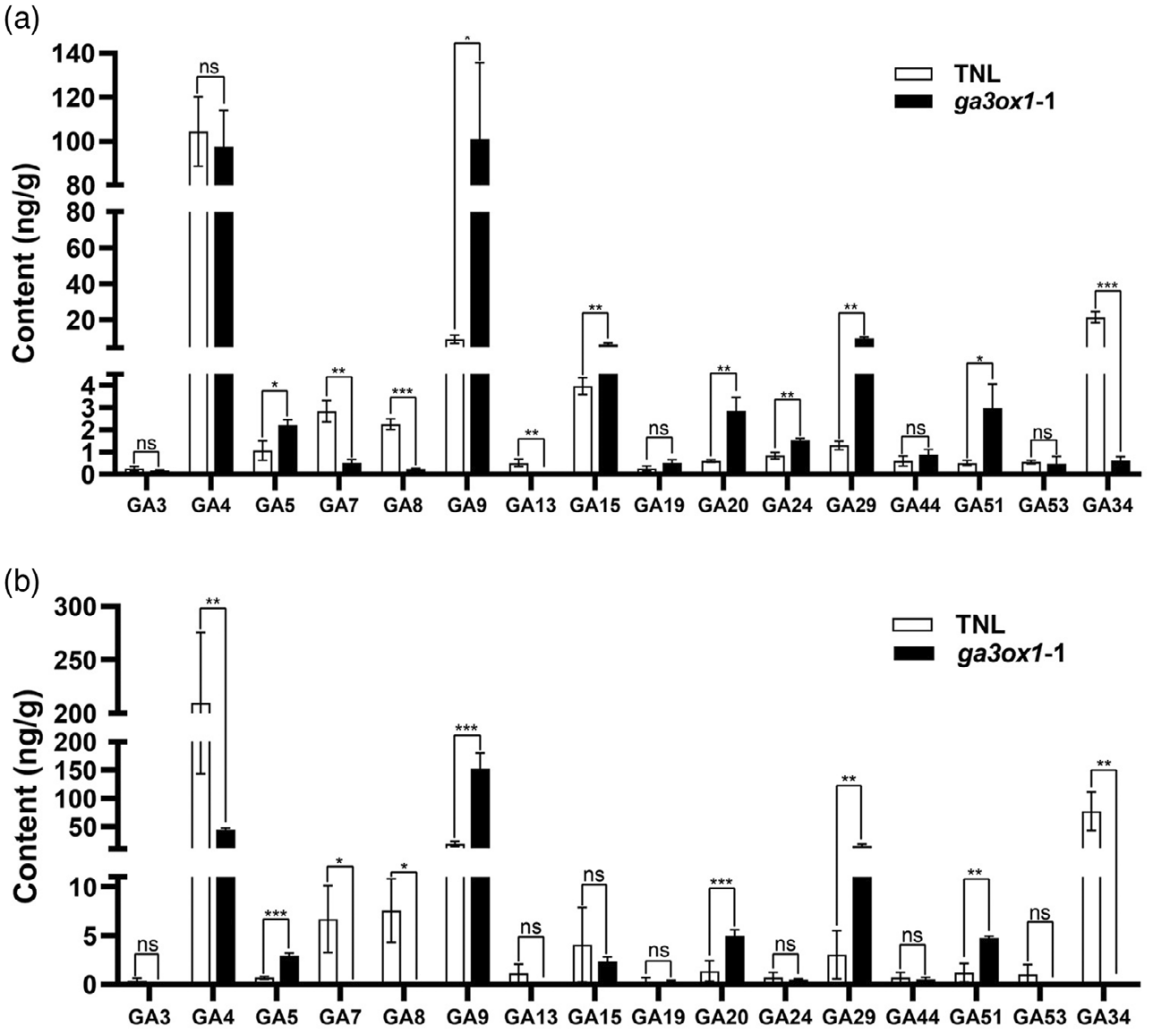
Comparison of GA isoform contents between *ga3ox1‐1* mutant and TNL. The GA isoform contents in the developing grain at (a) 10 days after pollination (10 DAP) and (b) 15 days after pollination (15 DAP) were compared between *ga3ox1‐1* and TNL. *Note*: Error bars indicate SD among three biological replicates. *P < 0.05, **P < 0.01, ***P < 0.001, ns, not significant, as determined by Student's *t*‐test. “TNL” indicates transgenic null line.

*HvGA3ox1* encodes an important enzyme in GA biosynthesis pathway (Pearce *et al*., 2015). We measured GA isoforms in the developing grains 10 and 15 days after pollination (DAP). We revealed vast differences in GA isoform compositions between *ga3ox1‐1* and TNL (Figure 6). The level of bioactive GA isoforms GA_7_ in *ga3ox1‐1* was significantly lower in the developing grains at 10 DAP than those in TNL (Figure 6a). The GA isoform profile in the developing grains of *ga3ox1‐1* mutant at 15 DAP had significantly lower bioactive GA_4_ and GA_7_, compared with those in TNL (Figure 6b). GA_4_ is the major bioactive GA isoform in the vegetative phase and involved in the regulation of growth and flowering transition (Sun *et al*., 2019; Zhang *et al*., 2014).

### Genes responsible for sucrose and carotenoid metabolism are downregulated in *ga3ox1‐1* mutant

**Figure 7:**
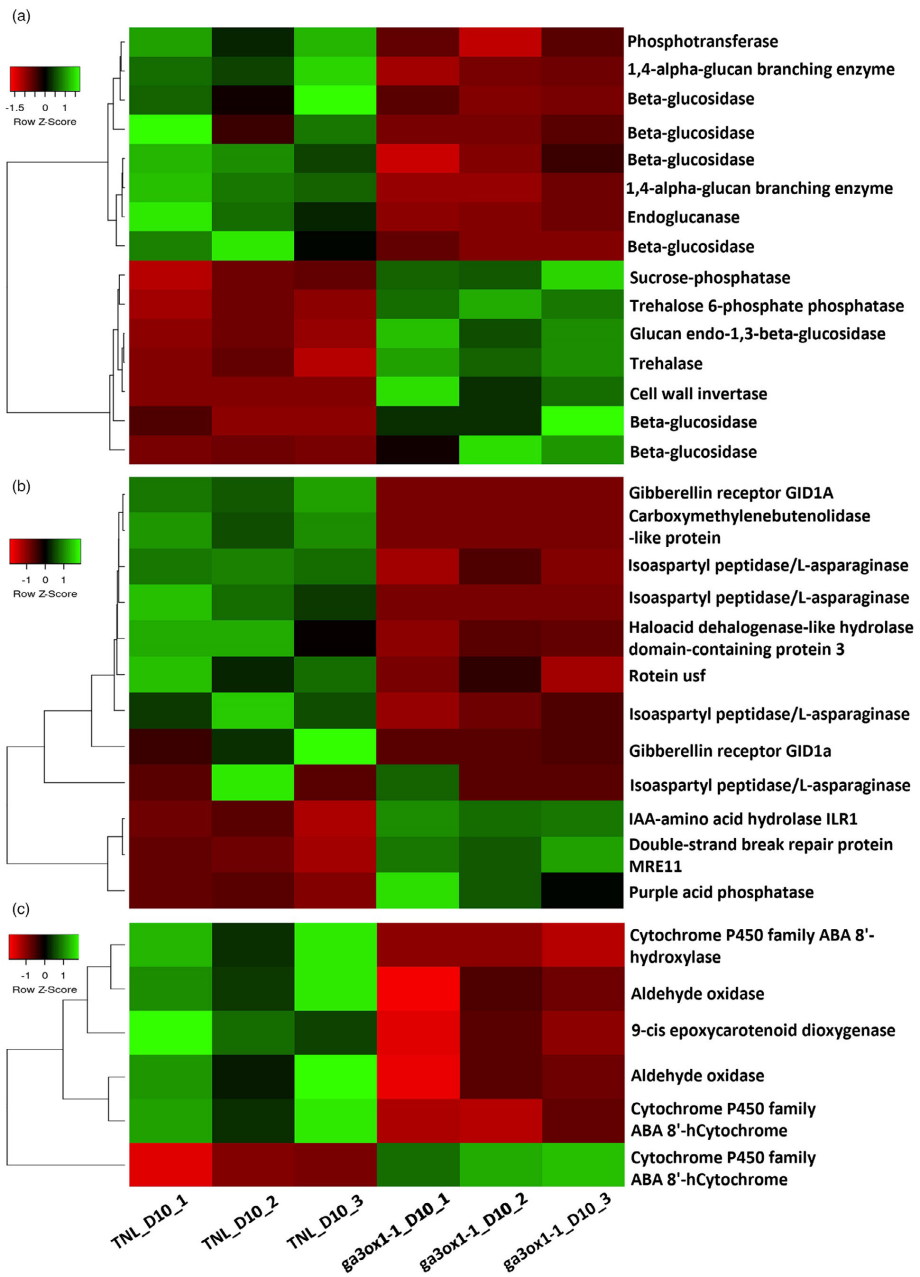
A heatmap illustrating significantly differentially expressed genes (DEG) involved in (a) starch and sucrose metabolism, (b) hydroxylase activity as well as (c) carotenoid biosynthesis pathways between *ga3ox1‐1* and TNL. *Note*: “DEG” indicates differentially expressed genes; “TNL” indicates transgenic null line; “D10” indicates 10 days after pollination. The colour (from green to red) indicates gene expression intensity from high to low.

To dissect the molecular mechanism of *HvGA3ox1* in regulating seed germinating, we investigated the differentially expressed genes (DEGs) in the developing grains at 10 DAP between TNL and *ga3ox1* mutants. A total of 1411 DEGs were identified in the developing grains at 10 DAP between *ga3ox1* mutants and TNL, including 620 up‐regulated and 791 down‐regulated DEGs. These DEGs were involved in a large array of pathways as identified by Kyoto Encyclopedia of Genes of Genomes (KEGG) (Figure 7). The top six significantly enriched KEGG pathways include starch and sucrose metabolism including 15 DEGs (Figure 7a). Compared to TNL, two genes encoding 1,4‐alpha‐glucan branching enzyme (HORVU.MOREX.r3.7HG0751650, HORVU.MOREX.r3.7HG0751660) and four genes encoding Beta‐glucosidase (HORVU.MOREX.r3.5HG0490890, HORVU.MOREX.r3.3HG0296310, HORVU.MOREX.r3.7HG0717390 and HORVU.MOREX.r3.3HG0218760), were downregulated in *ga3ox1‐1* mutant. In addition, there were 12 DEGs associated with hydroxylase activity (Figure 7b), including GIBBERELLIN‐INSENSITIVE DWARF1 (GID1) receptor gene (HORVU.MOREX.r3.4HG0344590 and HORVU.MOREX.r3.2HG0119270).

Besides the starch and sucrose metabolism and hydroxylase activity, carotenoid biosynthesis pathways were also significantly downregulated with five DEGs, one of which encoding 9‐cis epoxycarotenoid dioxygenase involved in Abscisic acid (ABA) biosynthesis (HORVU.MOREX.r3.6HG0607370) (Figure 7c).

## Discussion

Actively growing tissues contain high levels of bioactive GA, promoting cell division and expansion. Most semi‐dwarf crop varieties grown today carry one of a few variants that reduce the sensitivity to the plant hormone gibberellin (GA) leading to shorter statures, which has enabled the modern fertilization practices since the Green Revolution (Eshed and Lippman, 2019; Hedden, 2003; Peng *et al*., 1999). However, reduced GA level in semi‐dwarf crops can be accompanied by several adverse effects, including a short coleoptile. Coleoptile length is a key agronomic trait in many cereal crops and determines the maximum depth at which the seed can be sown. Long coleoptiles are linked to improved seedling establishment under water‐limited growing conditions (Luo *et al*., 2020). Excessively reduced plant height is not compatible with modern agronomic practices. Shorter coleoptile due to reduced levels of bioactive GA, discount yield potential from short stature (Pearce, 2021). Breeders are seeking alternative semi‐dwarfing genes with the potential to reduce plant height at an optimal height and increase yields while maintaining longer coleoptiles and greater early vigour. In this study, we characterized barley *GA3ox1* as a promising alternative semi‐dwarfing gene that reduces barley plants less than the currently used *sdw1* gene while increasing coleoptile length, which could provide essential adaptation to maintain yield under climate change. Moreover, we revealed that altered *GA3ox1* activity could increase seed dormancy to an ideal level, a feature beneficial to the malting industry.

### Barley *ga3ox1* mutants moderately reduce plant height

Plant height is one of the most important agronomic traits that impact grain yield and is considered a significant crop breeding target (Nadolska‐Orczyk *et al*., 2017). Altered plant height affects crop architecture, abiotic stress tolerance, lodging resistance and mechanical harvest (Liu *et al*., 2018). In barley, *sdw1/denso* (*HvGA20ox2*) is the widely used semi‐dwarfing gene in breeding programmes. *sdw1.d* is an allele of *sdw1/denso* successfully introduced into numerous barley varieties with a plant height reduction of approximately 20–30 cm (Dang *et al*., 2020; Kandemir *et al*., 2022; Teplyakova *et al*., 2017). The dramatically reduced plant height in varieties carrying *sdw1.d* is believed to improve the lodging resistance thus maintaining yield in heavily fertilized growing conditions. However, semi‐dwarf crops become too short for efficient mechanical harvest in some regions (Budak *et al*., 1995). Here, we show that altered *HvGA3ox1* activities reduce plant height, but to a less extent than currently used *swd1.d*. The natural *HvGA3ox1* haplotype Hap5 conferred a plant height approximately 10 cm shorter than other haplotypes, representing a promising alternative semi‐dwarfing allele for barley breeding. The dwarfing mutants, *ga3ox1‐1* and *ga3ox1‐2*, generated by CRISPR/Cas9 technology, reduced barley height approximately by 5 cm, supporting the mildly reduced GA signals as the result of altered *HvGA3ox1* activities. Meanwhile, altered *HvGA3ox1* activities showed no adverse impact on TKW and yield components. By contrast, *sdw1/denso* alleles, including *sdw1.d*, reduce TKW, thus negatively impacting grain yield (Kandemir *et al*., 2022; Mickelson and Rasmusson, 1994). Meanwhile, we observed different phenotypic effects of the *ga3ox1‐3* with 268 bp deletion, compared to the *ga3ox1‐1* with 4 bp deletion and *ga3ox1‐2* with 8 bp deletion. This result resembles the different phenotypic effects of semi‐dwarfing gene *sdw1.d* with 7 bp deletion and *sdw1.a* with the total deletion of *HvGA20ox2* (Jia *et al*., 2009; Xu *et al*., 2017).

### *HvGA3ox1* is associated with a coleoptile length

Coleoptile length is an important agronomic trait in barley and other cereal crops such as wheat, as it determines the maximum depth for the seed to be sown into the soil. Cultivars with short coleoptiles may fail to emerge because the first internode cannot elongate enough to bring the crown to the soil surface (Takahashi and Takeda, 1999). Most currently semi‐dwarf cultivars grown globally have relatively short coleoptiles (Chono *et al*., 2003; Hedden, 2003; Zhang, 2001). Developing cereal crop varieties with long coleoptile, allowing deep sowing to take advantage of stored soil water, has been advocated as one of the key adaptation strategies for production improvement under climate change with unreliable rainfall and increased drought stress (Zhao *et al*., 2022). Altered *GA3ox1* activities could increase coleoptile length despite reducing plant height, as we showed the increased coleoptile length in *ga3ox1* mutants.

The interaction of different phytohormones, facilitated by *HvGA3ox1*, may play a role in regulating coleoptile length. Abscisic acid (ABA) derived from carotenoids acts upstream and antagonism of the ethylene‐signalling pathway to control coleoptile growth in rice (Yin *et al*., 2015). Our RNA‐seq analysis showed that the three genes (*HORVU.MOREX.r3.7HG0743370*, *HORVU.MOREX.r3.7HG0743390* and *HORVU.MOREX.r3.6HG0607370*) encoding key enzymes involved in carotenoid biosynthesis pathway and ABA biosynthesis pathway were downregulated in the developing grains, likely by the disruption of *HvGA3ox1*. Meanwhile, we show that significantly upregulated *HvEIN2* (*HORVU.MOREX.r3.5HG0467950*) in *ga3ox1* mutant had 79% similarity with *OsEIN2* (*LOC_Os07g06130*). It is known that *OsEIN2* acts as a positive regulator of ethylene signalling in stimulating coleoptile length (Ma *et al*., 2013). Although further research needs to elaborate on the precise mechanism of how *ga3ox1* promotes the elongation of coleoptile in barley, it is known that plant height and coleoptile length appear to be largely under independent genetic control among GA‐sensitive wheat (Rebetzke *et al*., 2013). This paves the way for selecting *HvGA3ox1* alleles to develop barley varieties with short height and longer coleoptile varieties in barley.

### Delayed seed germination in *HvGA3ox1* mutants

Barley is the primary cereal used in the production of malt globally. The malting industry requires high‐quality grains with an optimal level of dormancy that is tolerant to pre‐harvest sprouting while ready to germinate without long storage requirements (Woonton *et al*., 2005). We show that altered *HvGA3ox1* alleles reinforce seed dormancy, which could provide better tolerance to pre‐harvest sprouting in future breeding practices, especially for the countries such as China, USA, Japan and Australia that are likely to have continuous rainy and humid weather during harvest (Tai *et al*., 2021). However, we observed that the dormancy weakened in 2 months after harvest, believed to be an ideal level of dormancy required by the malting industry for economical operation, whilst maintaining high malting quality (Woonton *et al*., 2005).

Our results suggested that the increased dormancy in *HvGA3ox1* mutants is associated with altered starch and sucrose metabolism in the developing grains (Figure 7). The expression of six genes involved in starch and sucrose metabolism pathway was downregulated in *ga3ox1* mutant compared to TNL, including two genes encoding a 1,4‐alpha‐glucan branching enzyme (HORVU.MOREX.r3.7HG0751650 and HORVU.MOREX.r3.7HG0751660). A study reported that the 1,4‐alpha‐glucan branching enzyme catalyses the last step in polysaccharide biosynthesis, which determines the structure of starch and glycogen in maize (Jia *et al*., 2013), providing an explanation of the late maturation in the *ga3ox1* mutant. We discovered additional downregulated genes encoding beta‐glucosidase (HORVU.MOREX.r3.5HG0490890, HORVU.MOREX.r3.3HG0296310, HORVU.MOREX.r3.7HG0717390 and HORVU.MOREX.r3.3HG0218760) were previously reported to be involved in hydrolysis in rice (Akiyama *et al*., 1998). We found that GID1A and GID1a were downregulated in *ga3ox1* mutant. ID1a which acts as a gibberellin receptor is a positive regulator of seed germination and is involved in hydroxylase activity in *Arabidopsis thaliana* (Ge and Steber, 2018).

### Highly efficient gene editing in a commercial barley cultivar Vlamingh

Genotype dependency is a crucial factor in barley genetic transformation and severely limits the capacity for barley improvement using genome‐editing approaches. For example, tissue culture procedures are only developed and tested on a few genotypes (Han *et al*., 2011). The Scottish cultivar Golden Promise is the barley transformation reference and is the most used genotype for gene manipulation studies due to the ease of transformation (Harwood, 2012). Recently, new tissue culture systems have been developed to overcome this bottleneck, such as the modification of anther culture for gene editing in commercial barley cultivars (Han *et al*., 2021) and the application of growth‐regulating genes like *TaWOX5* (Wang *et al*., 2022), *Baby Boom* (*Bbm*) and *WUSchel 2* (*Wus2*) (Lowe *et al*., 2016). In the present study, the editing rate for *HvGA3ox1* was approximately 71% based on the donor, the Australian commercial barley cultivar “Vlamingh,” which is comparable to the editing rate reported in Golden Promise (Karunarathne *et al*., 2022). Vlamingh which shows a medium maturity has a high mutation frequency and high efficiency of generation of homozygous mutations in *T*_0_, thus an efficient gene‐editing platform and a valuable alternative barley transformation reference for barley research and breeding.

## Conclusion

Current widely used semi‐dwarfing genes have adverse effects on agronomic practice in mechanized farming systems and discount yield under the present climate change scenarios marked by unreliable rainfall and frequent drought. Breeders are, therefore, actively seeking alternative semi‐dwarfing genes with the potential to reduce plants to an optimal height and increase yields while maintaining longer coleoptiles and greater early vigour. We present *GA3ox1* as a potential alternative semi‐dwarfing gene, which only moderately reduces plant height. The altered *GA3ox1* gene activities induced by CRISPR/Cas9 changed levels of active GA isoforms and led to elongated coleoptiles in the mutant. Altered *GA3ox1* activity also increased seed dormancy to an ideal level that would benefit the malting industry. Finally, we demonstrate that CRISPR/Cas9 can enhance allelic diversity by directly engineering genetic variation in specific target genes, paving the way for developing additional alternative semi‐dwarfing genes and alleles.

## Materials and methods

### Plant material and phenotypic data

As described in Hill *et al*. (2021), 632 accessions consisting of 250 cultivars and 382 breeding and research accessions from 37 countries throughout Europe, Asia, North and South America, Africa and Australia were selected from over 4000 accessions preserved at the Western Crop Genetics Alliance at Murdoch University (Perth, Australia) to represent the diversity of phenology. This selection spanned the entire spectrum of cultivated barley, including two‐row (92%) and six‐row (8%) genotypes and winter (7%), spring (92%) and facultative (1%) growth habits.

As described in Hill *et al*. (2019a), 12 field experiments were conducted in 2015 and 2016 across multiple diverse environments in Western Australia (South Perth, Geraldton, Katanning, Merredin and Esperance). The experimental design for the field trial sites was performed as described previously (Hill *et al*., 2019a,b, 2021). Briefly, all regional field trials (partially replicated design) were planted in a randomized complete block design using plots of 1 m × 3 m laid out in a row–column format. Field trials in South Perth with limited space were conducted using a hill plot technique with a 40‐cm distance within and between rows. Seven control varieties were used for spatial adjustment of the experimental data.

Phenotypic measurements, including days to Z49, plant height and grain yield, were obtained in each plot per field experiment across a variety of environments in 2015 and 2016 (Hill *et al*., 2019a). Days to Z49 is calculated based on the number of days from sowing to 50% awn emergence above the flag leaf. Plant height is determined by calculating the average height of all plants per plot. Grain yield is estimated by grain mass in each plot and calculated grain yield (kg/ha).

### Genotypic data and variant effect prediction analysis

A previous custom target enrichment sequencing assay was used to identify mutations in *HvGA3ox1* (Hill *et al*., 2019b). The impact of each variant in *HvGA3ox1* on DNA product was predicted using the Ensembl Variant Effect predictor toolset (Ensembl Variant predictor web interface http://www.ensembl.org/vep).

### Functional variant analyses

Significant Indel‐based haplotypes (*P* value <0.05) were detected by two‐way analysis of variance (ANOVA) with LSD and Duncan test in SPSS Statistics version 16.0 (SPSS Inc., Chicago, IL) and considered as functional Indel‐based haplotypes compared with the reference haplotype. The identification of the regions with similarity and difference was performed using pairwise sequence alignment in DNAMAN version 8.0 to indicate the functional and structural changes between two biological protein sequences.

### Generation of transgenic barley plant and detection of edited mutations

The barley cultivar Vlamingh was used as material for *Agrobacterium*‐mediated transformation, which is an ideal transformation donor with a medium maturity and high transformation efficiency (transformation efficiency: 78.6%) (Wang *et al*., 2022). On the transformation vector, the single‐guide (sg)RNA of *HvGA3ox1* gene was driven by *TaU3* promoter and the *Cas9* by the maize (*Zea mays*) *ubi* promoter (Liu *et al*., 2019).

The detailed single‐guide (sg)RNA sequence of *HvG3ox1* was designed based on the respective PAM sites for CRISPR/SpCas9 system. The 20 nucleotides upstream of a PAM motif (5′‐NGG‐3′) for the CRISPR/SpCas9 was selected using the fast CRISPR target site identification tool (web interface: http://www.e‐crisp.org/). The expression vector was introduced into *Agrobacterium* strain C58C1 by triparental mating (Ditta *et al*., 1980).

Immature barley grains were collected 2 weeks post anthesis and sterilized with 75% ethanol followed by a sterile water washing. Then, immature barley embryos were isolated and Agrobacterium‐mediated transformation was performed to obtain transgenic plants following the protocols described by Zang *et al*. (2022). Leaf samples were collected from different tillers of candidate *T*_0_ transgenic mutant plants. Genomic DNA was extracted from the collected leaf samples using a FastPure Plant DNA Isolation Mini Kit (Vazyme Biotech Co. Ltd, Nanjing, China) for Sanger sequencing. The target gene *HvGA3ox1* was amplified using its specific primers: HvGA3ox1F (CACAACCGCTACTTCGACTTGG), HvGA3ox1R (AATTCCTCCATCACGCCACTGC). Different mutant types were identified by aligning the reference sequences.

### Seed germination, root and coleoptile measurement

Five replicates for *ga3ox1* mutants and TNL were used for phenotyping. During the grain fill stage, plant height was measured from the ground to the spike, excluding awns. Regarding yield components, spike length (cm) was measured from the base of the spike to the tip of the terminal spikelet (awns excluded). The tiller number per plant was determined at the jointing stage. The spike number per plant was determined by counting spikes at the physiology maturity stage. The kernel number per spike was counted as the mean of kernel number of all spikes per plant at the physiology maturity. Thousand kernel weight was determined using the weight of the 150 kernels from each plant. Only the seeds of *ga3ox1*‐1 and *ga2ox1*‐2 were used for the germination test and trait measurement as *ga3ox1*‐3 did not produce enough viable seeds to allow germination test.

Fifty seeds per line stored at −20 °C for 2 months after harvesting, including *ga3ox1* mutants and TNL, were germinated in the petri dish on wet filter paper in the dark. Three replicates were set up for determining the germination rate and measuring root length as well as coleoptile length. The germinating rate was recorded every day in 1 week (7 days). All root and coleoptile lengths were measured on the 7th day after germination.

Statistic comparison of the Student's *t*‐test was implemented with SPSS (SPSS Inc.). Statistical significance was taken up at *P* < 0.05.

### RNA‐seq and quantitative assays of GA isoforms

Three biological replicates of developing seeds were collected at 10 days after pollination (10 DAP) and 15 days after pollination (15 DAP) from *HvGA3ox1*‐knockout lines and transgenic null lines (TNL) in the Vlamingh genetic background. The RNA was extracted and sequenced by GrandOmics (Wuhan). Six Gb of transcriptome data for each sample from Illumina Higseq 4000 platform was obtained. Differentially expressed genes (DEGs), defined as ¦Fold Change¦ >2 and *P*‐value <0.05. A *P*‐adjusted (FDR) <0.05 was used as the threshold for significant enrichment for GO function and KEGG (Kyoto Encyclopedia of Genes and Genomes) pathway analysis. Genes were annotated based on Morex v3.

(Mascher *et al*., 2021). Gibberellin contents in developing grains at 10 DAP and 15 DAP from *HvGA3ox1*‐knockout lines and TNL were quantified as described by (Ma *et al*., 2015), where three biological replicates were made for each line.

### Data statistics

Data statistics was implemented with SPSS (SPSS Inc.). Statistic comparison was conducted with Students's *t*‐test.

## Authors contributions

CL, MZ, SS and JC perceived the project concept. JC, WK, HY, GG and XY conducted gene editing; JC, CH, TH and CL conducted on phenotypic measurements and performed data analysis. JC, TH, CH and CL wrote the paper with inputs from other authors.

## Conflict of interest

The authors have no conflicts of interest to declare.

## Supporting information


**Figure S1** Phylogenetic tree of *HvGA3ox1* and *GA3ox1* orthologs in other crops.

**Table S1** The impact of 52 variants in *HvGA3ox1* on DNA product using variant effect prediction (VEP).

**Table S2** Linkage of the 52 genetic variants in *HvGA3ox1*.
