# Supplementary figures and images for: New semi‐dwarfing alleles with increased coleoptile length by gene editing of gibberellin 3‐oxidase 1 using CRISPR‐Cas9 in barley (Hordeum vulgare L.)
Source: Plant Biotechnol J. 2023 Feb 8;21(4):806–18. doi: 10.1111/pbi.13998 (PMC10037138; doi:10.1111/pbi.13998)

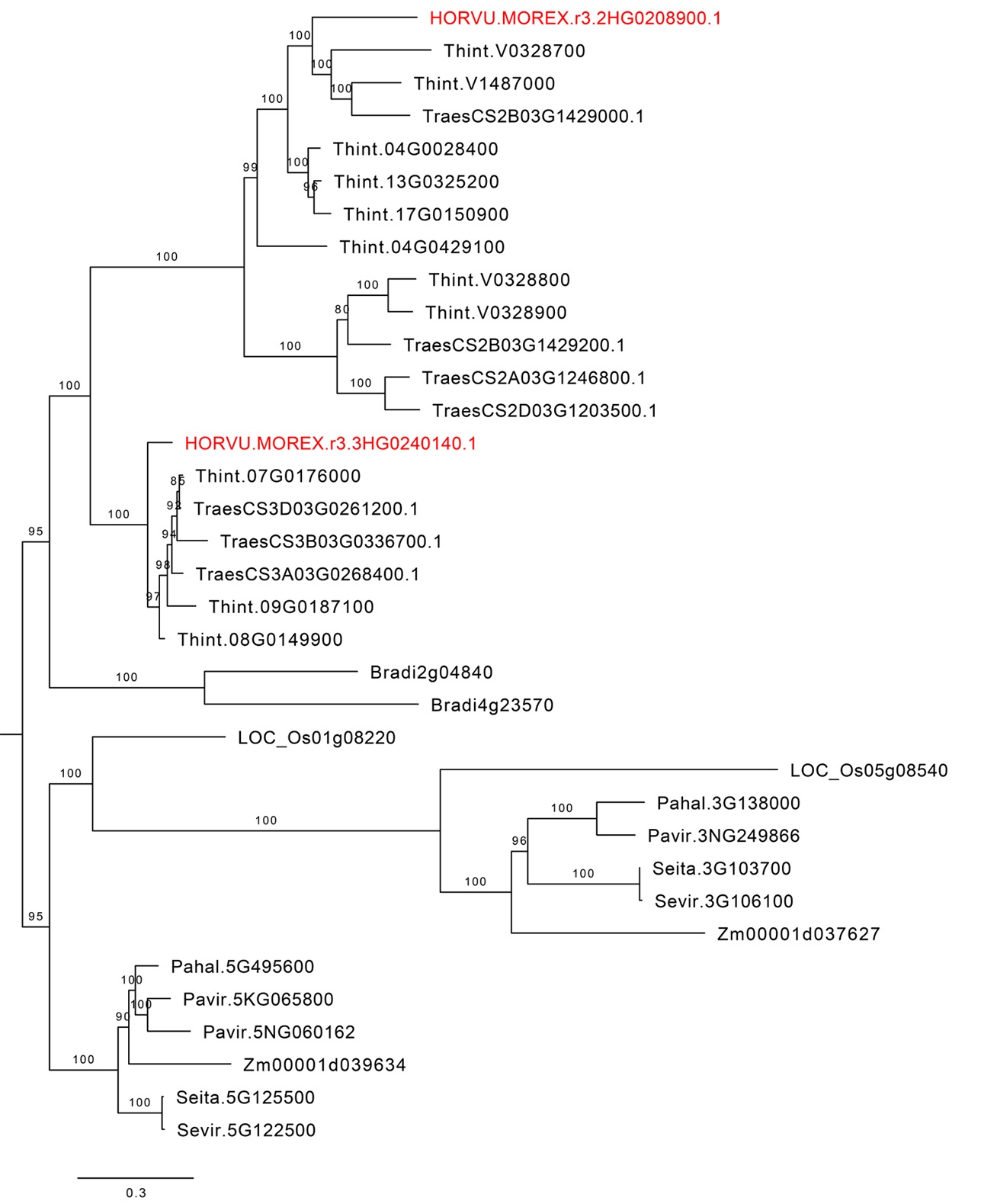

Supplement: Supplementary file 1 — Figure S1 Phylogenetic tree of HvGA3ox1 and GA3ox1 orthologs in other crops. [file PBI-21-806-s002.jpg]
